# De novo heterozygous variants of the *RSF1* gene are responsible for a syndromic neurodevelopmental disorder

**DOI:** 10.1038/s41431-026-02017-w

**Published:** 2026-01-28

**Authors:** Céline Jost, Tiffany Busa, Daniel Wegner, Marwan Shinawi, Elise Schaefer, Amélie Piton, Caroline Schluth-Bolard, Perrine Charles, Boris Keren, Katharina Mayerhanser, Theresa Brunet, Ulrich Schatz, Jennifer E. Neil, Christopher A. Walsh, Kathleen Sisco, Alexander J. Paul, Undiagnosed Diseases Network, Daniel Wegner, Chung Lee, Natalie Dykzeul, Devon Bonner, Jonathan A. Bernstein, Erin Sutcliffe, Ingrid M. Wentzensen, Catherine Froehlich, Kaleigh Liebler, Patricia Galvin Parton, Jody Weiss-Burns, Chloé Sagnol, Julian Delanne, Caroline Racine, Christel Thauvin-Robinet, Hana Safraou, Frédéric Tran Mau-Them, Yannis Duffourd, Ange-Line Bruel, Laurence Faivre

**Affiliations:** 1 Centre de Référence Anomalies du Développement et Syndromes Malformatifs de l’Interrégion Est, FHU TRANSLAD, Dijon University Hospital, Dijon, France; 2 Genetics Department, Timone Enfants University Hospital Center, Public Assistance-Marseille Hospitals, Marseille, France; 3 Department of Pediatrics, Washington University School of Medicine, St. Louis, MO USA; 4 Genetics Department, Public Strasbourg Hospitals, Strasbourg, France; 5 Genetic Diagnosis Laboratory, Strasbourg University Hospital, Strasbourg, France; 6 Laboratoire de Génétique Médicale, Institut de Génétique Médicale d’Alsace, INSERM UMRS_1112, Université de Strasbourg, Centre de Recherche en Biomédecine de Strasbourg, Strasbourg, France; 7 Genetic Department, Pitié-Salpêtrière Hospital, AP-HP, Sorbonne University, Paris, France; 8 School of Medicine, Institute of Human Genetics, Klinikum rechts der Isar, Technical University of Munich, Munich, Germany; 9 Division of Genetics and Genomics, and Howard Hughes Medical Institute, Boston Children’s Hospital, Boston, MA USA; 10 Departments of Pediatrics and Neurology, Harvard Medical School, Boston, MA USA; 11 Department of Pediatrics, Division of Medical Genetics, Stanford University, Palo Alto, CA USA; 12 GeneDx, LLC, Gaithersburg, MD USA; 13 Stony Brook Medicine, Lake Grove, NY USA; 14 Centre de Référence Déficiences Intellectuelles de Causes Rares, Dijon University Hospital Dijon, Dijon, France; 15 INSERM–University of Burgundy-UMR1231 GAD, Dijon, France; 16 Centre de Référence NeuroGène, Dijon University Hospital, Dijon, France; 17 Laboratoire de Génomique médicale–Centre NEOMICS, Dijon University Hospital, Dijon, France; 18 Centre de Référence GenoPsy, CHU Dijon Bourgogne, Dijon, France

**Keywords:** Next-generation sequencing, Neurodevelopmental disorders

## Abstract

Neurodevelopmental disorders (NDD) are a wide and heterogenous group of conditions due to impaired brain development, orchestrated by the crosstalk between genome and environment. Dynamic chromatin regulation during cortical development is fundamental, and chromatin remodelers are critical determinants of this process. Recently, numerous chromatin remodeling genes have been implicated in NDDs. By altering genes’ epigenetic state, mutated chromatin remodelers disrupt the spatiotemporal regulation of gene expression during development, potentially leading to severe consequences. The Remodeling and Spacing Factor 1 (*RSF1*) gene encodes a ubiquitous nuclear protein involved in chromatin remodeling, crucial for processes such as DNA transcription, replication, and repair. In this study, we identified by gene matching (*n* = 7) and literature search (*n* = 4) eleven unrelated individuals harboring de novo or inherited from a symptomatic parent heterozygous variants in *RSF1*. All individuals had an NDD, whether intellectual disability, autism spectrum disorder or developmental delay. From the seven individuals with detailed clinical information, unspecific and inconsistent associated features were described, including cranio-facial morphological features, musculoskeletal, digestive, vision, tone, epilepsy and brain MRI anomalies. Our data support the hypothesis that *RSF1* is important for brain development and a novel candidate gene for syndromic NDDs.

## Introduction

Over the past decade, exome (ES) and genome sequencing (GS) have become essential diagnostic tools for identifying genetic causes of neurodevelopmental disorders (NDD), given their extensive genetic heterogeneity and significant societal impact. Intellectual disability (ID) has a prevalence of ~2–3% in the general population [1], and its underlying origin is largely genetic. A meta-analysis revealed that ES accounts for an overall diagnosis rate of 42% (confidence interval: 35–50%) [2]. ES has therefore become a first-tier diagnostic test in most developed countries. Short-read GS makes it possible to establish additional diagnoses, but progress needs to be made to interpret non-coding variants [3]. The molecular bases remain unknown in about half of affected individuals and require the development of interpretation strategies to identify new disease-causing genes [4]. For example, trio-based analysis of ES/GS filtering of de novo variants, combined with data sharing, has been a successful approach to identifying candidate genes [5].

Several studies have shown an overrepresentation of causative variants in genes encoding chromatin remodelers in NDD cohorts [6, 7]. Chromatin remodeling genes play a crucial role, directly influencing the expression of genes required for brain development. Indeed, brain development begins at gastrulation and continues in the postnatal period, all of which is the result of fine and long-term spatio-temporal regulation of cell proliferation, differentiation and migration processes [7, 8]. Dysfunction of chromatin remodelers or remodeling complexes will impact the activation or repression of targeted genes through various mechanisms -epigenetic modulations (DNA methylation, histone methylation and/or histone acetylation), histone modifying enzymes, chromatin tridimensional organization changes, transcription regulation, and ATP-dependent chromatin remodelers and may result in NDD [6]. Many examples are well known: *CREBBP* (Rubinstein-Taybi syndrome MIM#180849) or *KANSL1* (Koolen-De Vries syndrome, MIM# 610443) involved in histone acetylation, *KDM6A* (Kabuki syndrome MIM#300867) or *KMT2A* (Wiedemann-Steiner syndrome MIM#605130) responsible for histone methylation, *MBD5* (ID MIM# 156200 or *CHD7* (CHARGE syndrome, #MIM214800) involved in DNA methylation. Variants in transcription factors such as *ZNF711* can lead to disorders like X-linked ID 97 (MIM#300803), while variants in three-dimensional chromatin loop organizers like *SMC3* or *SMC1A* are associated with Cornelia de Lange syndrome (MIM#610759 and MIM#300590, respectively). Wide chromatin remodeling complexes can also disturb gene expression, hydrolyzing ATP to produce energy that will disrupt the interaction between DNA and histones. The best-known is the switch/sucrose-non-fermenting (SWI/SNF) complex, which acts as a DNA translocase to destroy histone-DNA binding. Other ATP-dependent protein complexes are described and associated with different mechanisms: chromodomain-helicase-DNA binding (CHD) complexes, including nucleosome remodeling and deacetylase (NuRD) complex able to deacetylate targeted genes, inositol requiring 80 (INO80) complexes responsible for nucleosome eviction (INO80, SRCAP and TIP60/P400), and imitation switch (ISWI) complexes (NURF, CHRAC, ACF, CERF, WICH, NoRC, RSF) that impact nucleosome positioning in favor of heterochromatin formation and transcriptional repression [6, 9]. Numerous subunits of these chromatin remodeling complexes encoded by disease-causing genes are associated with syndromic or isolated NDD: *ARID1A* or *ARID2* (SWI/SNF complex) with Coffin-Siris syndrome (MIM#614607 and #617808), *CHD8* or *CHD5* (CHD complex) with syndromic ID (MIM#615032) and Parenti-Mignot syndrome (MIM#619873) respectively, and *YY1* (INO80 complex) with Gabriele-de Vries syndrome (MIM#617557).

The ISWI family is highly evolutionary conserved. In humans, sixteen ISWI complexes are known, including one ATPase subunit (SMARCA1 or SMARCA5) and one to three noncatalytic subunits: RSF-1/RSF-5 (SMARCA1/5 and RSF1), ACF-1/ACF-5 (SMARCA1/5 and BAZ1A), CHRAC-1/CHRAC-5 (SMARCA5/1, BAZ1A, CHRAC1 and POLE3), WICH-1/WICH-5 (SMARCA1/5 and BAZ1B), NoRC-1/NoRC-5 (SMARCA1/5 and BAZ2A), NuRF-1/NuRF-5 (SMARCA1/5, BPTF, RBBP7 and RBBP4), CERF-1/CERF-5 (SMARCA1/5 and CECR2) and BRF-1/BRF-5 (SMARCA1/5, BAZ2B). Certain subunits are strongly suspected of being involved in human pathology, such as SMARCA1, SMARCA5, BAZ1A, BAZ1B, BPTF and BAZ2B, which are associated with NDD [10–14], and BRF1, with cerebellofaciodental syndrome (MIM#616202).

The *RSF1* gene (Remodeling and Spacing Factor 1), also named *HBXAP* (Hepatitis B Virus X-Associated Protein (MIM#608522), is located at 11q14.1, comprises 16 exons and encodes a 1441 amino acid protein involved in the regulation of chromatin structure, ubiquitously expressed [15–17]. RSF1 functions as a histone chaperone within the RSF complex [18]. The role of RSF1 is poorly described, but it has been suggested as a candidate gene in ID and/or autism spectrum disorder (ASD) [19]. Several clinical manifestations observed in individuals with *RSF1* variants, including craniofacial malformations, ear and eye anomalies, pigmentary abnormalities, and endocrine or digestive dysfunctions, are suggestive of a partial neural crest deleterious effect for the missense variants. Chromatin remodeling genes, including ISWI family members, have been shown to regulate neural crest specification and migration [20]. Therefore, although *RSF1* has not yet been directly linked to neural crest biology, its function as an ISWI chromatin remodeler may similarly influence gene networks essential for neural crest-derived tissues.

Here, we report on *RSF1* as a novel gene in dominant NDD-causing chromatin remodelers. We describe developmental/neurodevelopmental features identified in seven novel unrelated individuals, adding to the four reported in the literature within large cohorts of NDDs/ASD/ID.

## Material and methods

### DNA sequencing

DNA was extracted from samples according to standard procedures. Trio or solo-based exome sequencing (ES) (individuals 1,3,5,6,7) or trio or solo-based GS (individuals 2,4) were performed following the manufacturer’s recommendations (Supplementary data).

### Sanger sequencing (if available)

Genomic DNA was amplified by polymerase chain reaction (PCR) using HotStarTaq PCR kits (Qiagen) according to the manufacturer’s protocol in all individuals and both parents. For Sanger sequencing, PCR products were purified by the Agencourt CleanSEQ system (Beckman Coulter) and sequenced with the BigDye Terminator Cycle Sequencing kit, v3.1 (Applied Biosystems) in the ABI 3730 sequencer (Applied Biosystems).

### Data sharing/identification of additional individuals

Individuals or guardians consented to data sharing. Data was shared using the Matchmaker Exchange platform GeneMatcher [21]. Additional de novo variants of the *RSF1* gene were found using the denovo-db and Decipher databases.

## Results

**Fig. 1:**
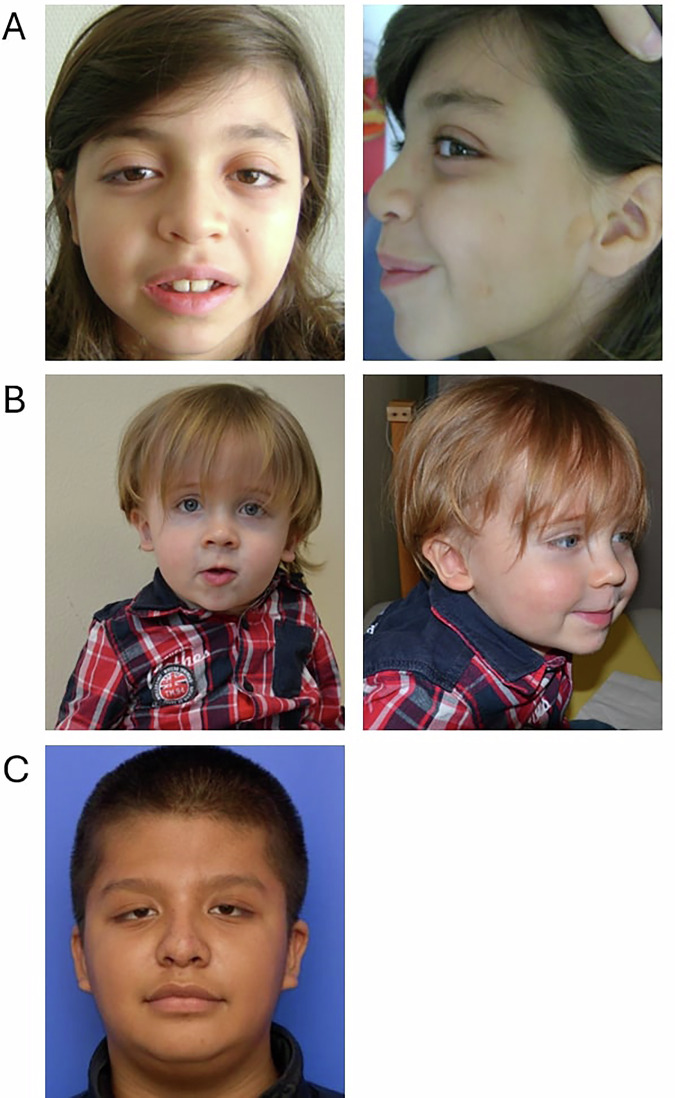
Photographs of individuals 1, 2 and 6. **A** Individual 1: Note the downslanting palpebral fissures, epicanthus, large nostrils, short columella, wide base of the nose, bulbous nose, thick vermilion of the lips, macrodontia and posteriorly rotated low-set ears. Note the scars from chondromas on the left cheeks and preauricular area. **B** Individual 2: Note the wide nostrils, high columella, wide base of the nose, concave bridge of nose, narrow mouth and thin upper lip. **C** Individual 6: Note the low hairline implantation, post frontal orbital advancement, palpebral slits oriented downwards and outwards, ptosis, epicanthus, low-set ears, bulbous nasal tip and thick vermilion of the lips.

Initially, individual 1 was diagnosed by ES as part of the DISSEQ project [22]. He had no family history. He was born at 40 weeks’ gestation by cesarean section after an unremarkable pregnancy. He had a height of 55 cm (99.72th percentile), weight of 3580 g (68.28th percentile) and head circumference of 36 cm (92.44th percentile). Clinical examinations revealed chondromas of the left cheek and preauricular area. The individual underwent surgery at ten months for a trigonocephaly discovered at eight months. He walked at thirteen months and spoke his first words at ten months. Later, learning disabilities at school prompted psychometric tests revealing an intellectual development disorder, with a total intellectual quotient that cannot be calculated with the WISC-V scale (VCI (verbal comprehension) 73, VSI (visuo-spatial) 57, FRI (fluid reasoning) 58). He had also undergone testicular surgery for cryptorchidism at the age of eight years. The following morphological features were observed: down slanting palpebral fissures, epicanthus, large nostrils, short columella, wide base of the nose, bulbous nose, thick vermilion of the lips, macrodontia and posteriorly rotated low-set ears (Fig. 1A). At the last visit at the age of 15 years, he was 174 cm tall (+ 0.3 SD), weighed 52 kg (–0.6 SD) and had a head circumference of 54 cm (–1SD). He had astigmatism, a few discreetly hyperpigmented spots on the left forearm and flat feet. He attended an adapted school, could read, write, and do small sums, but had difficulties with graphics. Paraclinical investigations included a normal brain CT scan and abdomen/renal ultrasound, as well as normal hearing tests. Fragile X testing and chromosomal microarray (CMA) were negative. ES identified de novo nonsense variant in *RSF1* (NM_016578.4) in the PHD-finger domain, chr11(GRCh38):g.77691163G>A, p.(Arg966Ter). The variant is absent from gnomAD (v4.1.0), with higher deleterious scores: CADD-Phred: 38.00, MPA: 10 (Table 1). The *RSF1* gene appears intolerant to haploinsufficiency (pLI:1, pLOEUF: 0.15, gnomAD (v4.1.0). No additional candidate variant was found.

**Table 1:** Clinical summary of *RSF1* individuals.

Table 1	Individual 1	Individual 2	Individual 3	Individual 4	Individual 5	Individual 6	Individual 7	Total
Sex	M	M	F	F	M	M	F	4 M, 3 F
Country of residence	France	France	USA	France (origin : Laos)	Germany	USA	USA	
Molecular features								
Method	ES	GS	ES	GS	ES	ES	ES	5 ES, 2 GS
Variant (GRCh38; NM_016578.4)	chr11:g.77691163 G > A c.2896 C > T	chr11:g.77667177 T > A c.4066 A > T	chr11:g.77693549 C > T c.2778 G > A	chr11:g.77693562C>T c.2765G>A	chr11:g.77678085C>A c.3133+1G>T	chr11: :g.77413521_77413528del c.748_755del	chr11:g.77394837C>A c.2983G>T	4 nonsense, 2 missense, 1 splice site
Variant NP_057662.3 (protein)	p.(Arg966Ter)	p.(Lys1356Ter)	p.(Met926Ile)	p.(Arg922His)	p.?	p.(Ser250AspfsTer4)	p.(Glu995Ter)	
Bioinformatic predictive scores	CADD: 38.00 MPA: 10	CADD: 38.00 MPA: 10	CADD: 26.70 PolyPhen-2 : 0.92 SIFT: 0.008	CADD: 28.50 PolyPhen-2 : 0.999 SIFT: 0.043	CADD: 34 spliceAI Donor lost: 100 SPIP: 98.41%	MPA: 10	CADD: 41.00 MPA: 10	
*Inheritance*	de novo	de novo	de novo	de novo	The healthy mother carries the variant with an allele fraction of ~20%	de novo	de novo	6 dn, 1 inherited from the mother with mosaic
*Clinical features*								
Age at last examination	15 y	6y7m	4 y	26 y	27 y	11y10m	14 y	Range 4–27 y
Weight (SD) Height (SD) OFC (SD)	52 kg (–0.6) 174 cm (+ 0,3) 54 cm(–1)	27,9 kg (+ 1.4) 126 cm (+ 1.2) 61 cm (+ 6)	14,5 kg (–0,5) 96,1 cm (–0.8) 45 cm (–2.9)	64 kg (+ 0.8) 175 cm (+ 1.8) 59 cm (+ 3.0)	170 cm (–0.9) NA NA	NA (+ 2.23) NA (–1.32) NA (+ 0.36) (9y9m)	44.1 kg (+ 0.53) 163.5 cm (–0.82) unknown	2 /6 macrocephaly 1/6 microcephaly
Upper face anomalies	Trigonocephaly	-	Low posterior hairline	-	Prominent forehead	Metopic craniosynostosis, Trigonocephaly, forehead remodeling, low hair implantation	-	3/7
Midface anomalies	Posteriorly rotated low-set ears, oblique downslanting palpebral fissures, epicanthus, large nostrils, bulbous nose with wide base, short columella, chondroma	Abnormal pinna morphology, small auditory canals, wide base of the nose with large nostrils, and high columella	Hypertelorism, synophrys, long eyelashes and palpebral fissures, depressed nasal bridge, short anteverted nose, low columella	-	Small auricles with folded helix edge, bushy eyebrows, slightly raised nose tip	Post frontal orbital advancement, oblique downslanting palpebral fissures, ptosis, epicanthus, low-set ears, bulbous nasal tip	-	5/7
Lower face anomalies	Thick vermilion, macrodontia	Prognathism, narrow mouth, thin upper lip	Retrognathia, long and smooth philtrum, thin upper lip, high arched palate	-	Retrognathia, prominent philtrum, thin upper lip	Dental caries, ectopically erupting tooth, thick vermilion of the lips	-	5/7
Cardiac anomalies	NA	-	Persistent left superior vena cava and asymptomatic PVCs	-	-	-	-	1/6
Gastrointestinal disturbance	-	Moderate feeding difficulties	Constipation	Abdominal meteorism	-	Fatty liver	-	4/7
Skin defects	Hyperpigmentation :left arm	-	Dry skin, hirsutism	-	Pterygium	Café au lait macules	Psoriasis; post-surgical	5/7
Extremities anomalies	Pes planus	Pes planus	Fifth finger clinodactyly, 4–5 toes clinodactyly, abnormal nails, cutaneous 2–3 syndactyly	-	Talipes equinovarus, pes planus, short tapering fingers	-	-	4/7
Endocrine/Urology anomalies	Cryptorchidy	-	-	-	Delayed puberty	Type 2 diabetes mellitus.	-	3/7
Ophthalmological anomalies	Astigmatism	-	Visual processing disorder	-	-	ptosis, right exotropia, anisometropia, amblyopia, myopia, astigmatism	-	3/7
Neurodevelopmental/neurological features								
Psychomotor/speech delays	-	+	+	+	-	+	-	4/7
ID	Mild ID	Results at the lower limit of normal	Mild ID	Moderate to severe ID	No formal testing	Suspected ID	No ID	3 mild ID, 1 moderate to severe ID, 1 within lower normal limit, 1 suspected, 1 no ID
Educational difficulties	Learning difficulties, writing difficulties	School for special needs	Short attention span, learning difficulties	School for special needs	Yes, behavioral abnormalities related to autism, but was able to finish training as an office administrator	In special education with IEP	IEP in school for focus on attention and social aspects of education.	7/7
Gait anomalies	-	-	+	-	NA	-	-	1/6
Seizures	-	-	+	+	-	-	-	2/7
Hypotonia, muscle weakness	-	+	+	-	-	-	-	2/7
Hyperlaxity	-	+	-	-	NA	+	-	2/6
ASD	-	+	- (but sensory seeking behaviors)	-	+	-	+	3/7
Abnormal MRIc	Normal brain CT scan (no MRI)	Ventriculomegaly, dilated subarachnoid spaces	-	Bilobed arachnoid cyst of the posterior fossa	NA (Not performed)	NA (Not performed)	NA (Not performed)	2/4
Other features								
Other			Wheezing with URIs; recurrent ear infectionsDrooling		Pterygium, wide-spaced nipples	Asthma	ADHD	
Other relevant variants				arr[GRCh37] 11p2(45804505_45960637)x1dn		Xq22.1q22.2 arr[hg19] Xq22.1q22.2(101596735_102648626)x2 mat SMAD6 p.(Asp292Asn)	TGFBR1 (c.1138G>T)	

*OFC* occipitofrontal circumference, *NA* Not available, dn de novo, *ASD* autism spectrum disorder, *ID* intellectual disability, *PVCs* Premature ventricular contractions, *URIs* upper respiratory tract infection.

International data-sharing via GeneMatcher identified five additional individuals with heterozygous *RSF1* variants, from France (2), Germany (1) and the USA (2) (Table 1 and Fig. 1).

Individual 2 had no family history. Macrocephaly was diagnosed antenatally. He was born at 39 weeks’ gestation, was 53 cm (98.15th percentile), weighed 4450 g (99.47th percentile), and had a head circumference of 39 cm (99.99th percentile). At birth, dysplastic ears with a small ear canal and prognathism were noticed. During infancy, the child had moderate feeding difficulties. He also had flat feet, hyperlaxity, hypotonia and muscle weakness. In terms of development, he showed severe language delay, with his first words at age three, and articulatory difficulties. A neuropsychological assessment revealed a profile at the lower limit of the norm with heterogeneous results (WPPSI-IV (VCI: 78; VSI; 75; FRI: 80; PSI: 59)). EEG is normal. Probable neurovisual disorder, attention difficulties, and ASD were suspected, which may have reduced the individual’s performance. At the last consultation at 6 years and 7 months, he weighed 27.9 kg (+ 1.4 SD), measured 126 cm (+ 1.2 SD), and had severe macrocephaly, with a head circumference of 61 cm (+ 6 SD). Some morphological features could be noted, such as wide nostrils, high columella, wide base of the nose, concave bridge of nose, narrow mouth and thin upper lip (Fig. 1B). He could only make simple sentences. Tests for Angelman syndrome, Prader-Willi, *PTEN* and a panel of 551 ID genes and metabolic health were normal. Paraclinical examinations, including cardiac ultrasound and electroencephalography, were normal. Brain MRI showed ventriculomegaly and dilatation of the subarachnoid spaces. GS revealed a de novo *RSF1* variant in chr11(GRCh38):g.77667177T>A, p.(Lys1356Ter) outside a functional domain, absent from gnomAD (CADD-Phred: 38.00, MPA: 10, Table 1).

**Fig. 2:**
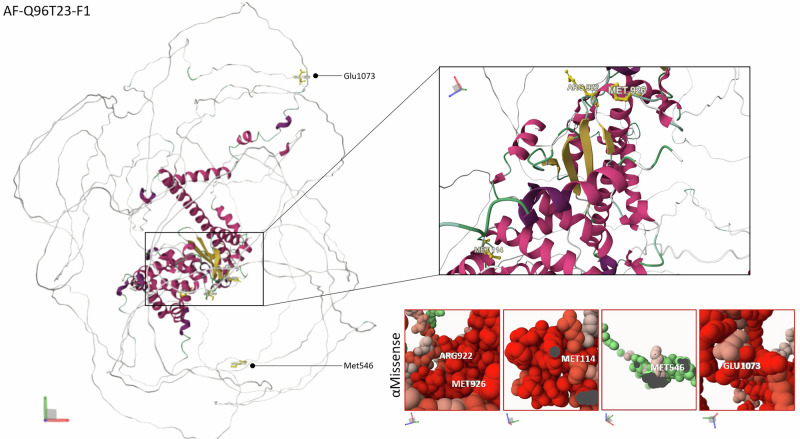
3D representation of the missense variants, mapped onto the predicted 3D structure of *RSF1* using the AlphaFold model (AF-Q96T23-F1). Amino acids at positions 922, 114, and 926 are located within β-sheet structures, with AlphaMissense predicting a deleterious effect. Amino acids 1073 and 546 lie outside the functional/structural core of RSF1, with a predicted deleterious effect for 1073 and a benign prediction for 546.

Individual 3 had no family history. She was born at 38 weeks’ amenorrhea, with normal birth measurements. No particular events were noted during the neonatal period. Sitting was acquired at nine months, walking at eighteen months and first words at 18 months, with language regression and delays in gross and fine motor skills. She developed seizures at nine months, mainly tonic–clonic and occasional episodes of staring. EEG showed bifrontal and generalized spike-wave discharges. Her epilepsy was controlled by three drugs: lamotrigine, cannabidiol and phenobarbital. At the age of four, she had only fifteen words with no associations and pronunciation difficulties. The individual was diagnosed with an ID without neuropsychological testing (based on clinical impression), receives physical, occupational and speech therapy and has an individualized education program at preschool. Morphologically, she had microcephaly with a head circumference of 45 cm (–2.97 SD) at four years, slight hypertelorism, long palpebral clefts, slight micro-retrognathism, low-lying columella, depressed nasal bridge, short anteverted nose, high arched palate, thin upper lip, long and smooth philtrum, synophrys and bushy eyebrows, long eyelashes, a low posterior hairline, and hirsutism on the back and limbs. She had clinodactyly of the fifth finger, with red/pink discoloration of all distal fingertips, hyperconvex nails and dystrophic toenails, mild cutaneous syndactyly of the second and third toes bilaterally, overlap of toe 2 on toe 1 and medial deviation of toes 4–5. The skin was dry. She has recurrent ear infections with normal hearing. There was also wheezing, drooling and an abnormal gait, with a tendency to swing her legs and invert her feet. She had hypotonia and muscle weakness. On the gastrointestinal side, the individual presented with constipation. She had a visual processing disorder. Cardiac evaluation revealed a persistent left superior vena cava and asymptomatic premature ventricular contractions. At the last examination at 4 years of age, she weighed 14.5 kg (–0.52 SD), was 96.1 cm tall (–0.84 SD), and her head circumference was 45 cm (–2.97 SD). Investigations comprised microcephaly panel sequencing, CMA, ES, mitochondrial DNA analysis and searches for Prader-Willi/Angelman syndromes, which did not display any candidate to explain the clinical features. Research reanalysis of ES revealed a de novo missense variant in the PHD-finger protein domain, chr11(GRCh38):g.77693549C>T, p.(Met926Ile), absent from gnomAD, localized within β-sheet structures (Fig. 2), with deleterious prediction scores (CADD-Phred: 26.70, PolyPhen-2: 0.92, SIFT: 0.008, Table 1, FoldX ∆∆G: 0.305, REVEL: 0.318, ESM1b: -10.78, AlphaMissense: 0.9972, Table 2). No additional candidate variant was identified by GS.

**Table 2:**
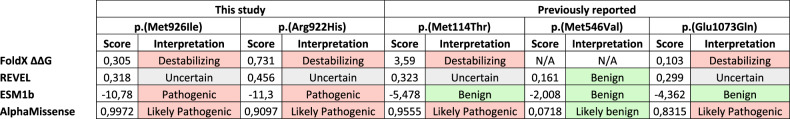
In silico prediction scores for *RSF1* missense variants.

In silico prediction scores for *RSF1* missense variants identified in this study (p.(Met926Ile), p.(Arg922His), p.(Met114Thr)) and previously reported variants (p.(Met546Val), p.(Glu1073Gln)). Four complementary prediction tools were used: FoldX ΔΔG (impact on protein stability), REVEL (meta-predictor of missense pathogenicity), ESM1b (deep-learning evolutionary constraint model), and AlphaMissense (AI-based structural classifier).

Color coding reflects the interpretation provided by each tool (pathogenic/ likely pathogenic in red, uncertain in gray, benign/likely benign in green).

Individual 4 had no family history. She was born at 41 weeks’ amenorrhea, with normal birth measurements. There were no particular events during the neonatal period. The first warning signs were developmental delay at nine months, with sitting acquired at eleven months, walking at twenty months and first words at three years. A first seizure occurred at the age of 5-6 years, in a febrile context, with a fall, eyes rolling back, teeth clenching, shaking, biting of the tongue, and eyes and head turned to the right, which lasted 20–25 min and stopped with valium. The second seizure came two months later, without a febrile context and lasted five minutes. The EEG was of little help. Treatment with sodium valproate was initiated following this episode. After several years without seizures and with normal EEGs, treatment was stopped. However, the seizures returned and lamotrigine was added to Sodium valproate. An EEG was carried out immediately afterwards and revealed a few slow theta-wave spikes in the left and right temporal areas. Later, after three years without seizures, the Sodium valproate was stopped for leukoneutropenia, and there was a recurrence of seizures on discontinuation, leading to the introduction of a Lamotrigine-levetiracetam combination. Over the next three years, there were episodes of confusion and disruption of contact, suggestive of convulsive seizures confirmed by EEG, as well as episodes suggestive of hallucinations. Treatment was increased, but the partial seizures persisted. Over the next four years, there were episodes of apraxia in dressing, axial stiffness, contact breakage with occasional loss of urine, followed by drowsiness and amnesia lasting a few minutes. The last EEG revealed a less well-constructed background pattern on the left, with abundant epileptic figures in the right temporal and sometimes fronto-temporal areas. At the last examination at 26 years, she was 175 cm tall (+ 1.81 SD), weighed 64 kg (+ 0.80 SD), and her head circumference was 59 cm (+ 3 SD). She had no other dysmorphic features except macrocephaly. Developmentally, she was able to form sentences, had delayed motor skills and a moderate to severe ID, with a total intellectual quotient of 40. She also had abdominal meteorism. *NSD1* testing was normal, as were standard karyotype and Fragile X testing. As part of the DEFIDIAG project [23], a de novo *RSF1* variant in a functional domain was found in chr11(GRCh38):g.77693562C>T, p.(Arg922His), absent from gnomAD, highly conserved across organisms, localized within β-sheet structures (Fig. 2), with deleterious prediction scores (CADD Phred: 28.50, PolyPhen-2: 0.999, SIFT: 0.043, Table 1, FoldX ∆∆G: 0,731, REVEL: 0,456, ESM1b: -11,3, AlphaMissense: 0,9097, Table 2). Another diagnosis was retained, that of a de novo 156Kb deletion arr[GRCh37]11p2(45804505_45960637)x1, including exons 16 to 19 out of 19 of *PHF21A* associated with syndromic intellectual developmental disorder (MIM #618725).

Individual 5 had no family history. No information was available on his delivery, neonatal period or early development except the diagnosis of clubfeet. The individual had mild ID (determined by clinical impression with no standardized test performed). Morphologically, he had a prominent forehead, bushy eyebrows, slightly raised nose tip, hypertelorism, prominent philtrum, retrognathism, small auricles with folded helix edge, narrow lips, pterygium and wide nipples. He also has talipes equinovarus. He had a late onset of puberty. He had a depressive episode during adolescence. He did not develop epilepsy. At the last examination at the age of 27 years, he was 170 m tall. He had a normal karyotype, Noonan gene panel, and CMA. ES found a variant in chr11(GRCh38):g.77678085C>A, NM_016578.3:c.3133+1G>T, absent from GnomAD database predicted to impact on splicing (CADD Phred: 34, SpliceAI Donor lost: 1.00, SPIP: alteration of the consensus splice site: risk 98.41%, Table 1). The parents' status was unknown.

Individual 6 had a family history of developmental disorders. One of his maternal half-brothers is developmentally delayed, and another is hyperactive. One of these siblings also suffers from kidney failure. A paternal half-sister has a short stature with a history of learning difficulties. A paternal half-brother has episodes of fainting/weakness, and another has unexplained infertility. He was born at 39 weeks’ amenorrhea after a pregnancy marked by insulin-requiring maternal gestational diabetes, with birth measurements unknown. There were no particular events during the neonatal period. Trigonocephaly was noted shortly after birth. Developmentally, he has a global developmental delay with sitting acquired at 7 months, walking at 24 months, first words at 4 years, a delay in expressive and receptive language, and a gross motor delay. He is in special education with an Individualized Education Plan (IEP). Total intellectual quotient could not be determined due to the young age at which neuropsychological evaluations were conducted. At 2 years 5 months, the Capute Scales results were as follows: CLAMS 64, CAT 96, and full scale DQ 80. At the last examination at 11 years and 10 months, height was +2.23 SD, weight –1.32 SD and OFC + 0.36 SD. His morphological features included low hairline implantation, post-frontal orbital advancement, forehead remodeling, palpebral slits oriented downwards and outwards, mild ptosis, epicanthus, right exotropia, low-set ears, bulbous nasal tip, thick vermilion of the lips, metopic craniosynostosis, dental caries, ectopically erupting tooth (Fig. 1C), and café au lait macules with irregular borders. He had hypermobility, mainly of the small joints; he could flex his thumb up to his forearm, as well as his fifth finger, to more than ninety degrees; his knees and arms appeared to be hyperextended, but he was unable to touch the ground with the palm of his hand. He had a history of mild anisometropia, refractive amblyopia, moderate myopia and astigmatism. He had type 2 diabetes mellitus, asthma and a fatty liver. Fragile X testing was normal. Other candidate variations were found in this individual: an Xq22.1q22.2 microduplication arr[hg19] Xq22.1q22.2(101596735_102648626)x2 of uncertain significance by array-CGH, and a heterozygous variant of uncertain significance in *SMAD6* (NM_005585: c.874G>A, p.(Asp292Asn)) by a panel of RASopathy genes and ES. Both are inherited from the asymptomatic mother. ES identified a de novo frameshift variant in *RSF1*, chr11:(GRCh38):g.77413521_77413528del, p.(Ser250AspfsTer4). The variant was absent from GnomAD (v4.1.0), with higher deleterious scores: MPA: 10 (Table 1).

Individual 7 had a family history of NDD, with ADHD reported in three maternal uncles and the maternal grandfather. She was born at 41.5 weeks’ amenorrhea following an uneventful pregnancy. Her birth measurements were 54.6 cm (98.90th percentile); 3560 g (49.79th percentile), and head circumference was not recorded. There were no notable complications during the neonatal period. Developmentally, limited early data were available. Independent walking was achieved at 12 months. She was diagnosed with ADHD and oppositional defiant disorder around age 7 years, and with ASD at 14 years and with generalized anxiety disorder and social phobia. There is no ID. On the WISC-V assessment, conducted at 14 years, she obtained a full-scale IQ of 104 (confidence interval 98–109), placing her in the average range (61st percentile). She attends school with an IEP focusing on attentional and social support. Language level at 14 years was age-appropriate. At her most recent clinical examination at 14 years, her weight was 44.1 kg (+ 0.53 SD), and height was 163.5 cm (–0.82 SD). No dysmorphic features were noted. Her medical history is notable for psoriasis, a post-surgical rash with delayed wound healing, and a methicillin-resistant Staphylococcus aureus (MRSA) infection at 14 years. She also presents with scoliosis of the thoracolumbar spine and pectus excavatum. Fragile X testing, karyotype analysis, and chromosomal microarray (array-CGH) were normal. ES identified a variant of uncertain significance in TGFBR1 (NM_004612.4: c.1138G>T) and a de novo nonsense variant in RSF1 at chr11:g.77394837C>A, resulting in a premature stop codon: p.(Glu995Ter). This variant was absent from gnomAD (v4.1.0) with higher deleterious scores: MPA: 10, CADD: 41.00 (Table 1).

To further explore the potential structural impact of these missense variants, we mapped their positions on the predicted 3D structure of RSF1 using the AlphaFold model (AF-Q96T23-F1). The variants are distributed without clear clustering but often localizing within conserved or structurally constrained regions (Fig. 2). This representation provides additional insights into possible variant sensitivity areas within RSF1, complementing the in silico predictions. Variants reported in this study show consistently deleterious profiles across several predictors, especially ESM1b and AlphaMissense, and are all located within structurally constrained regions of the protein, close to its predicted functional core. This structural positioning, together with convergent pathogenicity scores, supports their functional impact. Conversely, previously reported variants showed discordant or predominantly benign predictions, and their amino-acid positions lie further from the predicted 3D functional center of RSF1, in regions that appear less structurally constrained (Table 2).

## Discussion

NDDs are a heterogenous group of disorders, including ID, caused by variants, mainly de novo, in >1500 genes [24]. Wide national and international consortium projects using ES and GS largely contribute to gene identification, and data sharing through the GeneMatcher platform has been very successful [5, 25]. Launched in September 2013, GeneMatcher is a freely accessible web-based tool developed as part of the Baylor-Hopkins Center for Mendelian Genomics [21]. It was created with the goal of identifying additional individuals with rare phenotypes who had variants in the same candidate disease gene. A notable fraction of monogenic DD/ID syndromes share chromatin dysregulation as a patho-mechanism driving a general perturbation of transcription during development, especially in the brain, where we find cell-type specificity and diversity, resulting from finely controlled processes of growth, differentiation and cell fate decisions [26, 27]. Chromatin remodelers are crucial parts of cell information processing machinery, integrating external and internal signals into gene expression patterns, and defects in chromatin remodeling lead to a relaxation of epigenetic control [6]. *RSF1* encodes an ISWI family protein involved in nucleosome assembly and positioning, and consequently in replication of heterochromatin, organization of higher order chromosome structure, and transcriptional regulation [14]. RSF1 facilitates access to DNA during processes such as DNA replication, transcription and repair [28]. RSF1 is specifically enriched at mitotic centromeres, where it will regulate the dynamics of H2A histone modifications and mediate heterochromatin formation [29]. RSF1 forms a heterodimer with SMARCA5 (RSF-5 complex) to promote nuclear import and stabilize SMARCA5 [18]. RSF-5 complex seems to be predominantly expressed in early neuronal development in progenitor cells [14]. RSF1 can also interact with SMARCA1 [18], preferentially in differentiated neurons. Some members of the ISWI family are associated with NDDs: SMARCA1 and SMARCA5 pathogenic variants cause ID/DD [14, 30], BPTF is associated with Neurodevelopmental Disorder with Dysmorphic Facies and distal Limb anomalies (NEDDFL) syndrome (MIM# 617755), and BAZ1B is commonly deleted in Williams-Beuren syndrome (MIM# 194050). The phenotype described in humans is similar to that observed in KO (knock-out) mouse models with abnormal neuronal proliferation or differentiation, causing macrocephaly or cerebral hypoplasia, and various behavioral/developmental disorders [14]. Neuron-specific Rsf1 knockout mouse models displayed no neuropathological abnormalities but showed an absence of neuronal apoptosis triggered by excessive DNA strand breaks during neurogenesis [31]. In contrast to Smarca1/5, Rsf1 appears to be inessential for neurodevelopment in mice, but this hypothesis must be tempered by the possibility that other ISWI proteins compensate for Rsf1 deficiency during brain development [31]. In *Xenopus*, RSF1 plays the role of a reader of histone H2A lysine 119 ubiquitination, crucial in early development. The *rsf1* knockdown embryos showed neural and neural crest defects [32]. Differences in species-specific sensitivity to chromatin remodeling imbalance may also contribute, as variants in other ISWI subunits often produce severe neurodevelopmental phenotypes in humans or *Xenopus* but only mild or tissue-restricted effects in mice [33]. Finally, the neuron-specific Rsf1 knockout model restricts gene inactivation to postmitotic neurons, leaving Rsf1 expression intact in early progenitors, glial cells, and neural crest–derived lineages. Such restriction likely spares critical developmental processes, including brain patterning and craniofacial morphogenesis, that may depend on Rsf1 activity during early embryogenesis. Moreover, *RSF1* is over-expressed in many types of tumors, resulting in deregulation of DNA repair pathways, maintaining chromosome stability and suppressing the transcription of some oncogenes, facilitating tumor resistance to treatment [34].

Candidate variants in the *RSF1* gene have been reported in four isolated individuals in prior studies that investigated de novo variants in large ID/ASD cohorts [19, 35–37]. Additional clinical information from these cases reported herein includes microcephaly and ASD reported in Individual 10, as well as ID and dysmorphic features not otherwise specified in Individual 11, whose variant was inherited from a father with ID (Supplementary Table 1).

Here we report on seven novel individuals, bringing the number of those carrying a de novo *RSF1* variant and NDD to eleven, including five nonsense, five missense and one splice-site (Fig. 1). All individuals had a syndromic NDD, with ID or limit scores for 7/11, ASD for 4/11 and developmental delay for 6/11. The clinical features described include mild morphological anomalies of the nose (5/7) and lips (5/7, including thin and thick lips), abnormal head circumference (3/6, including macrocephaly or microcephaly), ear anomalies (3/7), digestive manifestations (4/7), abnormal columella (3/7), feet anomalies (3/7, including pes planus and talipes equinovarus), abnormal chin (3/7, including retrognathism or prognathism), vision anomalies (3/7), eyebrows anomalies (2/7), hypotonia (2/7), epilepsy (2/7) and cerebral MRI anomalies (2/4). The presence of another diagnosis may explain the more severe intellectual features of individual 4, but the exact roles of either cannot be precisely determined. A dual diagnosis is not rare in ID/DD cohort, which can represent around 3.5% of the cohort [38]. Mainly, the phenotype presentation, including craniofacial dysmorphism, ear and eye anomalies, pigmentary changes, endocrine and digestive dysfunctions, as well as musculoskeletal and neurodevelopmental features, suggests the involvement of neural crest cells during early development as has been demonstrated in Xenopus [32].

**Fig. 3:**
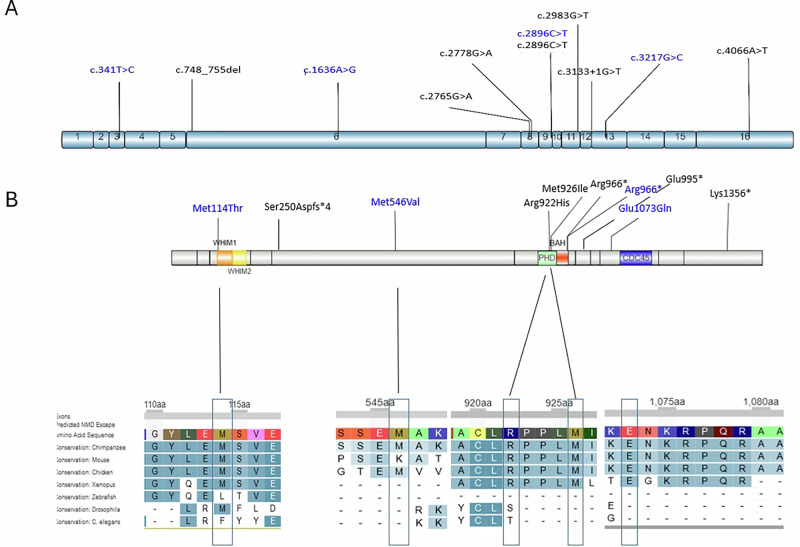
Graphical representation of the *RSF1* structure and identified variants. **A** Schematic representation of the *RSF1* protein. **B** Schematic representation of RSF1 cDNA. *In blue: variants in the literature. In black: variants identified in this study*. Made by IBS 2.0 [40].

The *RSF1* gene is predicted to be intolerant to loss-of-function (LoF) variants (pLI=1) (gnomAD, v4.1.0), supporting the loss-of-function mechanism for truncating variants. Phosphorylation sites in the C-terminal region of RSF1 (Ser1359 and Ser1375) are crucial for PLK1 recruitment, and consequently chromosome alignment, segregation and heterochromatin formation during mitosis. The loss of these active sites could explain why deletions of the last exon of RSF1 also impact the function of the protein [39]. However, the impact of missense variants remains difficult to assess. Missense variants located in the WHIM and PHD domains are in high missense-constrained regions that are highly evolutionary conserved (Fig. 3). PHD is a small domain essential to recruit transcription factors and nucleosomes. The structure of *RSF1* is poorly understood in silico modeling of missense variants remains difficult to interpret. Physicochemical-based prediction scores may support or argue against the deleterious nature of the identified variants, but these remain predictions based on a protein that is still insufficiently characterized. This highlights the need for functional studies, including approaches such as episignature analysis.

As chromatin remodelers play a direct role in gene expression and epigenetic mechanisms, looking for DNA methylation alteration appears as one possible functional test of disease-causing variants. Numerous mutated chromatin remodeling genes exhibit specific DNA methylation patterns in NDDs [40]. Overall, we postulate here that *RSF1*, a gene implicated in the regulation of chromatin remodeling, could be responsible for a novel syndromic NDD. Further descriptions are needed to better define the clinical spectrum associated with pathogenic *RSF1* variants.

## Supplementary information

Supplementary figure

Supplementary_data

## Data Availability

Additional data are available from the corresponding author on reasonable request.

## References

[1] Totsika V, Liew A, Absoud M, Adnams C, Emerson E. Mental health problems in children with intellectual disability. Lancet Child Adolesc Health. 2022;6:432–44. 35421380 10.1016/S2352-4642(22)00067-0

[2] Sánchez-Luquez KY, Carpena MX, Karam SM, Tovo-Rodrigues L. The contribution of whole-exome sequencing to intellectual disability diagnosis and knowledge of underlying molecular mechanisms: a systematic review and meta-analysis. Mutat Res Rev Mutat Res. 2022;790:108428. 35905832 10.1016/j.mrrev.2022.108428

[3] van der Sanden BPGH, Schobers G, Corominas Galbany J, Koolen DA, Sinnema M, van Reeuwijk J, et al. The performance of genome sequencing as a first-tier test for neurodevelopmental disorders. Eur J Hum Genet EJHG. 2023;31:81–8. 36114283 10.1038/s41431-022-01185-9 PMC9822884

[4] Robinson PN, Krawitz P, Mundlos S. Strategies for exome and genome sequence data analysis in disease-gene discovery projects. Clin Genet. 2011;80:127–32. 21615730 10.1111/j.1399-0004.2011.01713.x

[5] Bruel AL, Vitobello A, Mau-Them FT, Nambot S, Duffourd Y, Quéré V, et al. 2.5 years’ experience of GeneMatcher data-sharing: a powerful tool for identifying new genes responsible for rare diseases. Genet Med J Am Coll Med Genet. 2019;21:1657–61. 10.1038/s41436-018-0383-z 30563986

[6] Mossink B, Negwer M, Schubert D, Nadif Kasri N. The emerging role of chromatin remodelers in neurodevelopmental disorders: a developmental perspective. Cell Mol Life Sci. 2021;78:2517–63. 33263776 10.1007/s00018-020-03714-5 PMC8004494

[7] D’Souza L, Channakkar AS, Muralidharan B. Chromatin remodelling complexes in cerebral cortex development and neurodevelopmental disorders. Neurochem Int. 2021;147:105055. 33964373 10.1016/j.neuint.2021.105055 PMC7611358

[8] Larrigan S, Shah S, Fernandes A, Mattar P. Chromatin remodeling in the brain-a NuRDevelopmental odyssey. Int J Mol Sci. 2021;22:4768. 33946340 10.3390/ijms22094768 PMC8125410

[9] Aydin ÖZ, Vermeulen W, Lans H. ISWI chromatin remodeling complexes in the DNA damage response. Cell Cycle Georget Tex. 2014;13:3016–25. 10.4161/15384101.2014.956551 PMC4615051 25486562

[10] Zaghlool A, Halvardson J, Zhao JJ, Etemadikhah M, Kalushkova A, Konska K, et al. A role for the chromatin-remodeling factor BAZ1A in neurodevelopment. Hum Mutat. 2016;37:964–75. 27328812 10.1002/humu.23034 PMC6681169

[11] Lalli MA, Jang J, Park JHC, Wang Y, Guzman E, Zhou H, et al. Haploinsufficiency of BAZ1B contributes to Williams syndrome through transcriptional dysregulation of neurodevelopmental pathways. Hum Mol Genet. 2016;25:1294–306. 26755828 10.1093/hmg/ddw010

[12] Stankiewicz P, Khan TN, Szafranski P, Slattery L, Streff H, Vetrini F, et al. Haploinsufficiency of the chromatin remodeler BPTF causes syndromic developmental and speech delay, postnatal microcephaly, and dysmorphic features. Am J Hum Genet. 2017;101:503–15. 28942966 10.1016/j.ajhg.2017.08.014 PMC5630163

[13] Scott TM, Guo H, Eichler EE, Rosenfeld JA, Pang K, Liu Z, et al. BAZ2B haploinsufficiency as a cause of developmental delay, intellectual disability, and autism spectrum disorder. Hum Mutat. 2020;41:921–5. 31999386 10.1002/humu.23992 PMC7262739

[14] Picketts D, Mirzaa G, Yan K, Relator R, Timpano S, Yalcin B, et al. Pathogenic variants in SMARCA1 cause an X-linked neurodevelopmental disorder modulated by NURF complex composition. Res Sq. 2023;29:rs.3.rs-3317938. 10.1038/s41467-025-64838-5 PMC12603178 41213919

[15] Loyola A, Huang JY, LeRoy G, Hu S, Wang YH, Donnelly RJ, et al. Functional analysis of the subunits of the chromatin assembly factor RSF. Mol Cell Biol. 2003;23:6759–68. 12972596 10.1128/MCB.23.19.6759-6768.2003 PMC193931

[16] Oppikofer M, Bai T, Gan Y, Haley B, Liu P, Sandoval W, et al. Expansion of the ISWI chromatin remodeler family with new active complexes. EMBO Rep. 2017;18:1697–706. 28801535 10.15252/embr.201744011 PMC5623870

[17] Shamay M, Barak O, Shaul Y. HBXAP, a novel PHD-finger protein, possesses transcription repression activity. Genomics. 2002;79:523–9. 11944984 10.1006/geno.2002.6717

[18] Li Y, Gong H, Wang P, Zhu Y, Peng H, Cui Y, et al. The emerging role of ISWI chromatin remodeling complexes in cancer. J Exp Clin Cancer Res. 2021;40:346. 34736517 10.1186/s13046-021-02151-x PMC8567610

[19] Bruno LP, Doddato G, Valentino F, Baldassarri M, Tita R, Fallerini C, et al. New candidates for autism/intellectual disability identified by whole-exome sequencing. Int J Mol Sci. 2021;22:13439. 34948243 10.3390/ijms222413439 PMC8707363

[20] Hu N, Strobl-Mazzulla PH, Bronner ME. Epigenetic regulation in neural crest development. Dev Biol. 2014;396:159–68. 25446277 10.1016/j.ydbio.2014.09.034 PMC4261016

[21] Sobreira N, F S. GeneMatcher: a matching tool for connecting investigators with an interest in the same gene. Hum Mutat. 2015;36:928–30. 26220891 10.1002/humu.22844 PMC4833888

[22] Soilly AL, Robert-Viard C, Besse C, Bruel AL, Gerard B, Boland A, et al. Cost of exome analysis in patients with intellectual disability: a micro-costing study in a French setting. BMC Health Serv Res. 2023;23:386. 37085862 10.1186/s12913-023-09373-z PMC10120135

[23] Binquet C, Lejeune C, Faivre L, Bouctot M, Asensio ML, Simon A, et al. Genome sequencing for genetic diagnosis of patients with intellectual disability: the DEFIDIAG study. Front Genet. 2021;12:766964. 35178068 10.3389/fgene.2021.766964 PMC8845475

[24] Leblond CS, Le TL, Malesys S, Cliquet F, Tabet AC, Delorme R, et al. Operative list of genes associated with autism and neurodevelopmental disorders based on database review. Mol Cell Neurosci. 2021;113:103623. 33932580 10.1016/j.mcn.2021.103623

[25] Hamosh A, Wohler E, Martin R, Griffith S, Rodrigues EDS, Antonescu C, et al. The impact of GeneMatcher on international data sharing and collaboration. Hum Mutat. 2022;43:668–73. 35170833 10.1002/humu.24350 PMC9133194

[26] Larizza L, Finelli P. Developmental disorders with intellectual disability driven by chromatin dysregulation: Clinical overlaps and molecular mechanisms. Clin Genet. 2019;95:231–40. 29672823 10.1111/cge.13365

[27] Kleefstra T, Schenck A, Kramer JM, van Bokhoven H. The genetics of cognitive epigenetics. Neuropharmacology. 2014;80:83–94. 24434855 10.1016/j.neuropharm.2013.12.025

[28] Min S, Choi YW, Yun H, Jo S, Ji JH, Cho H. Post-translational regulation of the RSF1 chromatin remodeler under DNA damage. Mol Cells. 2018;41:127–33. 29385673 10.14348/molcells.2018.2244 PMC5824022

[29] Lee HS, Lin Z, Chae S, Yoo YS, Kim BG, Lee Y, et al. The chromatin remodeler RSF1 controls centromeric histone modifications to coordinate chromosome segregation. Nat Commun. 2018;9:3848. 30242288 10.1038/s41467-018-06377-w PMC6155007

[30] Li D, Wang Q, Gong NN, Kurolap A, Feldman HB, Boy N, et al. Pathogenic variants in SMARCA5, a chromatin remodeler, cause a range of syndromic neurodevelopmental features. Sci Adv. 2021;7:eabf2066. 33980485 10.1126/sciadv.abf2066 PMC8115915

[31] Min S, Kim K, Kim SG, Cho H, Lee Y. Chromatin-remodeling factor, RSF1, controls p53-mediated transcription in apoptosis upon DNA strand breaks. Cell Death Dis. 2018;9:1079. 30348983 10.1038/s41419-018-1128-2 PMC6197202

[32] Parast SM, Yu D, Chen C, Dickinson AJ, Chang C, Wang H. Recognition of H2AK119ub plays an important role in RSF1-regulated early Xenopus development. Front Cell Dev Biol. 2023;11:1168643. 37529237 10.3389/fcell.2023.1168643 PMC10389277

[33] Goodwin LR, Picketts DJ. The role of ISWI chromatin remodeling complexes in brain development and neurodevelopmental disorders. Mol Cell Neurosci. 2018;87:55–64. 29249292 10.1016/j.mcn.2017.10.008

[34] Cai G, Yang Q, Sun W. RSF1 in cancer: interactions and functions. Cancer Cell Int. 2021;21:315. 34147108 10.1186/s12935-021-02012-9 PMC8214769

[35] Deciphering Developmental Disorders Study. Prevalence and architecture of de novo mutations in developmental disorders. Nature. 2017;542:433–8. 28135719 10.1038/nature21062 PMC6016744

[36] DECIPHER v11.30: mapping the clinical genome [Internet]. [cité 27 mars 2025]. Disponible sur: https://www.deciphergenomics.org/.

[37] De Rubeis S, He X, Goldberg AP, Poultney CS, Samocha K, Cicek AE, et al. Synaptic, transcriptional and chromatin genes disrupted in autism. Nature. 2014;515:209–15. 25363760 10.1038/nature13772 PMC4402723

[38] Racine C, Denommé-Pichon AS, Engel C, Tran Mau-Them F, Bruel AL, Vitobello A, et al. Orphanomix Physician's Group. Multiple molecular diagnoses in the field of intellectual disability and congenital anomalies: 3.5% of all positive cases. J Med Genet. 2023;61:36–46. 10.1136/jmg-2023-109170 37586840

[39] Lee HS, Park YY, Cho MY, Chae S, Yoo YS, Kwon MH, et al. The chromatin remodeller RSF1 is essential for PLK1 deposition and function at mitotic kinetochores. Nat Commun. 2015;6:7904. 26259146 10.1038/ncomms8904 PMC4918322

[40] Levy MA, McConkey H, Kerkhof J, Barat-Houari M, Bargiacchi S, Biamino E, et al. Novel diagnostic DNA methylation episignatures expand and refine the epigenetic landscapes of Mendelian disorders. HGG Adv. 2022;3:100075. 35047860 10.1016/j.xhgg.2021.100075 PMC8756545

