# Supplementary figures and images for: De novo heterozygous variants of the RSF1 gene are responsible for a syndromic neurodevelopmental disorder
Source: Eur J Hum Genet. 2026 Jan 28;34(4):554–64. doi: 10.1038/s41431-026-02017-w (PMC13046748; doi:10.1038/s41431-026-02017-w)

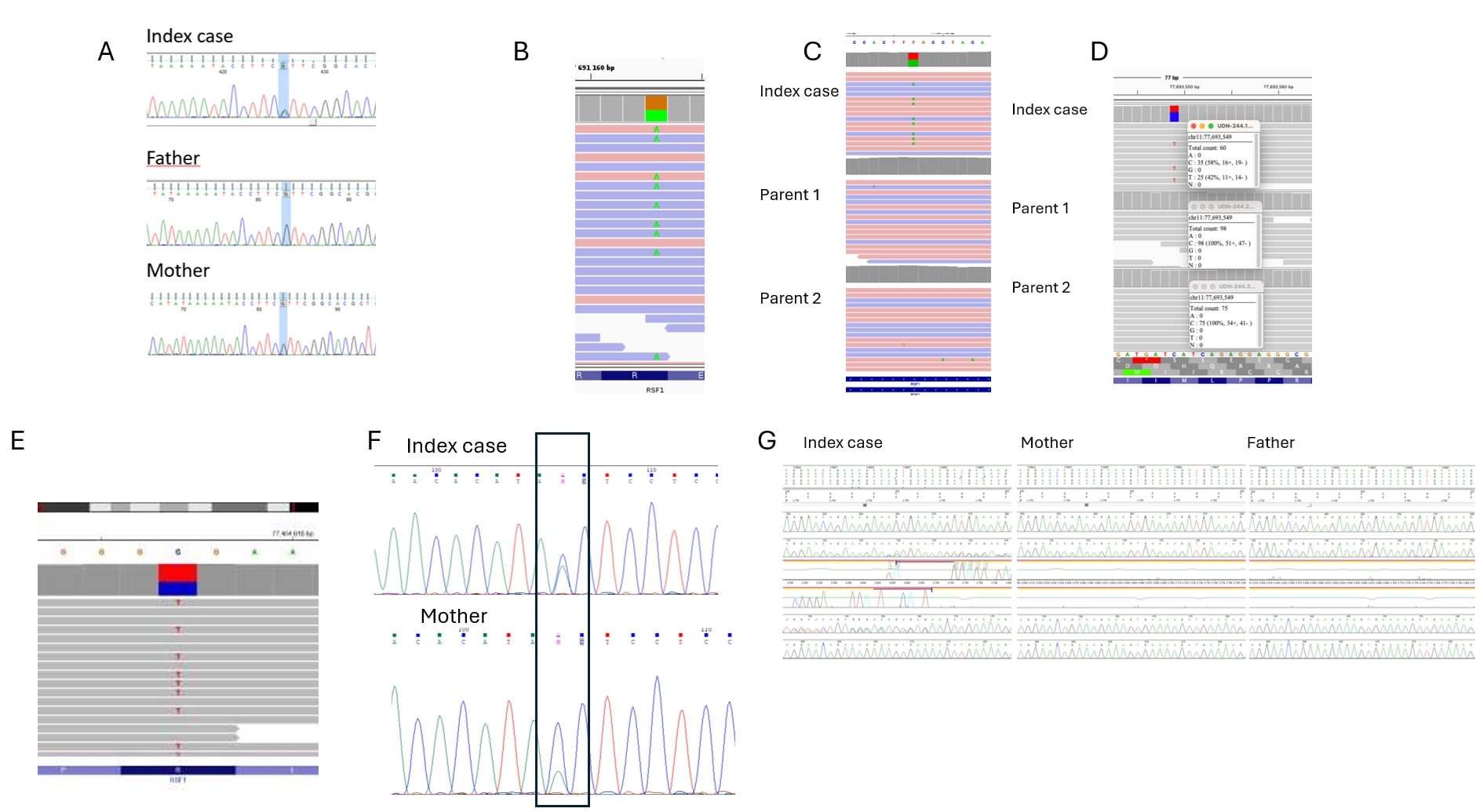

Supplement: Supplementary file 1 — Supplementary figure [file 41431_2026_2017_MOESM1_ESM.jpg]
