# Supplementary material for: De novo heterozygous variants of the RSF1 gene are responsible for a syndromic neurodevelopmental disorder
Source: Eur J Hum Genet. 2026 Jan 28;34(4):554–64. doi: 10.1038/s41431-026-02017-w (PMC13046748; doi:10.1038/s41431-026-02017-w)
Supplement: Supplementary file 2 — Supplementary_data [file 41431_2026_2017_MOESM2_ESM.docx]

**Supplementary data**

**Methods**

For individual 1, DNA was extracted from peripheral whole-blood samples, using the QIAamp DNA Mini Kit (Qiagen) following standard procedures. Exome capture and sequencing were performed at IntegraGen SA from 1 µg of genomic DNA per individual using the TWIG kit. The resulting libraries were sequenced on a HiSeq 4000 (Illumina) according to the manufacturer’s recommendations for paired end 76 bp reads. Obtained reads were aligned to the human genome reference sequence (GRCh37/hg19 assembly) using Burrows-Wheeler aligner (BWA; version 0.7.15). Duplicate reads were marked using Picard MarkDuplicates (version 2.4.1) (http://broadinstitute.github.io/picard/) and aligned read were then processed using GATK BaseRecalibrator and PrintReads (Genome Analysis Toolkit; version 3.8) to recalibrate base quality scores, according to GATK Best Practices. Quality control was performed on all BAM files by calculating depth of coverage via the RefSeq database (release 2018-11-11) with GATK DepthOfCoverage. SNPs and indels were identified from BAM files using GATK HaplotypeCaller. All variants were annotated using SnpEff (version 4.3).

For individual 2, trio GS was performed on the AURAGEN platform, as part of the French Genomic Medicine Initiative. Genomic DNA extracted from whole blood, using the QIAsymphony DNA Midi Kit, was sequenced according to standard procedures on a NovaSeq6000 instrument (Illumina). Sequencing data were aligned to the GRCh38.p13 reference genome using BWA-MEM (0.7.17). Variants were called by several algorithms including GATK GenotypeGVCF (4.1.8.0), Manta (1.6.0) and CNVnator (0.4.1), then filtered and annotated using Variant Effect Predictor (version 98.3), CuraGen and CuteVariant.

For individual 3, WUSTL RSF1 methods

Trio clinical exome sequencing (ES) was performed from whole blood derived DNA at GeneDx (Gaithersburg, MD), using a proprietary capture system developed by GeneDx for next generation sequencing with CNV calling, and sequenced on an Illumina platform. 98.7 % of the target region was covered at 10×, with a mean depth of 114×. Reads were aligned to the human reference genome build GRCh37 (hg19), and variants were analyzed with XomeAnalyzer software.

Trio clinical genome sequencing (GS) was performed from saliva swab derived DNA at PerkinElmer (Pittsburgh, PA), using 2x150 bp paired-end reads and sequenced on an Illumina platform. The genome was sequenced to an average of ≥50× coverage, and over 99 % of the disease causative gene target bases were covered at 10× coverage. Reads were aligned to the human reference genome build GRCh38 (hg38), and variants were analyzed with Illumina’s DRAGEN Bio-IT platform (version 2.03) and PerkinElmer’s internal ODIN (v1.01) software.

Research reanalysis was performed on the raw exome and genome data obtained from GeneDx and PerkinElmer, respectively. We followed Genome Analysis Toolkit (GATK) best practices for alignment (https://gatk.broadinstitute.org) of the ES and GS data to the human reference genome build GRCh38. We used GATK to jointly call single nucleotide (SNV) and indel variants and annotated them with ANNOVAR. We used a multi-caller then merge approach to call structural variants (SVs), using two read depth and two split read callers (Manta, Smoove, CNVnator, and CNVkit). SURVIVOR was used to merge the calls into a single file, and AnnotSV was used to annotate SVs. We prioritized SVs identified by multiple callers and/or that impact coding or splice regions. We called known Short Tandem Repeats (STRs) using Expansion Hunter.

For individual 4, the variant was identified through trio genome sequencing as part of the DEFIDIAG research project, with the methodology previously published.^1^

For individual 5, DNA fragments were enriched using the Twist Human Exome 2.0 Plus with Comprehensive Exome Spike-in and Mitochondrial Panel, and subsequently sequenced on the NovaSeq6000 (Illumina) platform, achieving an average coverage of more than 46x as 100 base pair paired-end reads. More than 98% of the target sequences had a minimum coverage of 20x. After preprocessing the sequencing data with Illumina bcl2fastq, adapter sequences were removed using cutadapt. The reads were then aligned to the Human Genome Assembly GRCh37 (hg19) using the Burrows-Wheeler Aligner (BWA). PCR duplicates and optical duplicates were removed with Picard MarkDuplicates. Variant calling was performed using various software tools: single-nucleotide variants (SNVs) were called with the Genome Analysis Toolkit 4 (GATK 4.2.3.0) and samtools, indels up to 20 bp were detected with Pindel, and copy number variations (CNVs) with ExomeDepth. Filtering and prioritization of the detected DNA variants were performed using the in-house software EVAdb (github.com/mri-ihg/EVAdb) and gnomAD.

For individual 6, using genomic DNA from the proband and parents, the exonic regions and flanking splice junctions of the genome were captured using the Twist Bioscience Exome 2.0 (Twist Biosciences, South San Francisco, CA). Massively parallel (NextGen) sequencing was done on an Illumina system with 150bp paired-end reads. Reads were aligned to human genome build GRCh37/UCSC hg19 and analyzed for sequence variants using a custom-developed analysis tool. Reported variants were confirmed, if necessary, by an appropriate orthogonal method in the proband and in selected relatives. Additional sequencing technology and variant interpretation protocol has been previously described^2^. The general assertion criteria for variant classification are publicly available on the GeneDx ClinVar submission page ( <http://www.ncbi.nlm.nih.gov/clinvar/submitters/26957/>).

**Bibliography**

1. Binquet C, C L. Genome Sequencing for Genetics Diagnosis of Patients With Intellectual Disability: The DEFIDIAG Study. *Front Genet*. 2022;12(766964).

2. Retterer K, Juusola J, Cho MT, et al. Clinical application of whole-exome sequencing across clinical indications. *Genet Med Off J Am Coll Med Genet*. 2016;18(7):696-704. doi:10.1038/gim.2015.148

|  | Individual 8  (Bruno LP, 2021 Dec) | Individual 9  (Decipher Developmental Disorders Study , 2017 Feb) | Individual 10  (Silvia De Rubeis, 2014 Nov) | Individual 11  (Decipher) |
| --- | --- | --- | --- | --- |
| **Cohort** | ID/ASD | DD | ASD | ID |
| **Number of individuals in the cohort** | 60 | 7580 | 3871 | NA |
| **Identified by** | ES | ES | ES | NA |
| **Variant (GRCh38)**  **NM_016578.4**  **NP_001356.1** | chr11:g.77676916C>G  c.3217 G>C  p.(Glu1073Gln) | chr11:g.77701593T>C c.1636A>G  p.(Met546Val) | chr11:g.77747067A>G  c.341T>C  p.(Met114Thr) | chr11:77691163G>A  c.2896C>T  p.(Arg966Ter) |
| **Bioinformatic predictive scores** | SIFT 0.006  PolyPhen 2 : 1.0  CADD Phred 26.1 | SIFT 0.618  PolyPhen 2 : 0.0  CADD Phred :1.32 | SIFT 0.103  PolyPhen 2 : 0.999  CADD Phred 25.50 | NA |
| **Allele count in GnomAD v4.1.0** | 1 | 2 | 0 | 0 |
| ***Inheritance*** | dn | dn | dn | Inherited from symptomatic father |
| ***Family history*** |  |  | Parents and grandparents are first cousins.  Sister : microcephaly, global developmental delay (no RSF1 variant)  Paternal first cousin: severe ID | Both parents have degrees of ID |
| ***Other*** |  | Other diagnosis | Homozygous VUS in *BCKDK* (c.375+3A>G) (MIM#614923) | Paternally inherited VUS in *MAP3K* (c.14_16dup) |
| ***Clinical data*** | NA | NA | Microcephaly, ASD | ID, dysmorphism |

**Table S1: Summary of the molecular and clinical data on four individuals in the literature.** ID: intellectual disability; DD: developmental disorder; ASD: autism spectrum disorder; NA: not available; dn: de novo;
